# Navigation system for robot-assisted intra-articular lower-limb fracture surgery

**DOI:** 10.1007/s11548-016-1418-z

**Published:** 2016-05-28

**Authors:** Giulio Dagnino, Ioannis Georgilas, Paul Köhler, Samir Morad, Roger Atkins, Sanja Dogramadzi

**Affiliations:** 1 Bristol Robotics Laboratory, University of the West of England, Coldharbour Lane, Bristol, BS16 1QY UK; 2 Bristol Royal Infirmary, Upper Maudlin Street, Bristol, BS2 8HW UK

**Keywords:** Medical robotics, Fracture surgery, Computer-assisted surgery, Fracture reduction planning, Image guidance, 3D medical imaging

## Abstract

**Purpose:**

In the surgical treatment for lower-leg intra-articular fractures, the fragments have to be positioned and aligned to reconstruct the fractured bone as precisely as possible, to allow the joint to function correctly again. Standard procedures use 2D radiographs to estimate the desired reduction position of bone fragments. However, optimal correction in a 3D space requires 3D imaging. This paper introduces a new navigation system that uses pre-operative planning based on 3D CT data and intra-operative 3D guidance to virtually reduce lower-limb intra-articular fractures. Physical reduction in the fractures is then performed by our robotic system based on the virtual reduction.

**Methods:**

3D models of bone fragments are segmented from CT scan. Fragments are pre-operatively visualized on the screen and virtually manipulated by the surgeon through a dedicated GUI to achieve the virtual reduction in the fracture. Intra-operatively, the actual position of the bone fragments is provided by an optical tracker enabling real-time 3D guidance. The motion commands for the robot connected to the bone fragment are generated, and the fracture physically reduced based on the surgeon’s virtual reduction. To test the system, four femur models were fractured to obtain four different distal femur fracture types. Each one of them was subsequently reduced 20 times by a surgeon using our system.

**Results:**

The navigation system allowed an orthopaedic surgeon to virtually reduce the fracture with a maximum residual positioning error of $0.95 \pm 0.3\,\hbox {mm}$ (translational) and $1.4^{\circ } \pm 0.5^{\circ }$ (rotational). Correspondent physical reductions resulted in an accuracy of 1.03 ± 0.2 mm and $1.56^{\circ }\pm 0.1^{\circ }$, when the robot reduced the fracture.

**Conclusions:**

Experimental outcome demonstrates the accuracy and effectiveness of the proposed navigation system, presenting a fracture reduction accuracy of about 1 mm and $1.5^{\circ }$, and meeting the clinical requirements for distal femur fracture reduction procedures.

**Electronic supplementary material:**

The online version of this article (doi:10.1007/s11548-016-1418-z) contains supplementary material, which is available to authorized users.

## Introduction

In the surgical treatment for lower-leg intra-articular fractures, the fragments have to be positioned and aligned to reconstruct the fractured bone as precisely as possible (anatomical reduction) [1], to allow the joint to function correctly again [2], avoiding post-operative chronic pain, a reduced functioning of the limb, arthritis, and as a consequence, potential (partial) disablement [3, 4].

Currently, the treatment for lower-limb joint fractures consists in anatomical surgical reduction and rigid internal fixation, involving an open incision into the joint, manual reduction in the fracture, and fixation using a metallic plates and screws, or intramedullary nails [5]. Although this open procedure can be effective, it is associated with extensive damage to the soft tissues, slow bone healing, and increased risk of infection, with consequent prolonged hospitalization, rehabilitation time, and health-related costs [6, 7]. Minimally invasive surgical techniques (i.e. percutaneous) have been developed to mitigate the problems related with open surgery. These techniques involve fragment manipulation using pins inserted in the fragments through small incisions in the patient’s flesh. Such techniques are associated with a faster recovery and a lower risk of infection compared to open surgery techniques [8]. However, the major challenge in minimally invasive fracture surgery (MIFS) using the current surgical set-up is to deduce the desired reduction position of bone fragments from multiple intra-operative fluoroscopic images of the fracture. The 2D nature of these images, the localized and limited 2D field of view, and their low resolution do not provide enough information to the surgeon in respect of the fracture alignment and rotation—which is essentially a three-dimensional problem—possibly causing a misinterpretation of the corrective parameters. In fact, optimal pose correction of the articular surface in 3D requires restoring six parameters: three translations and three rotations [3]. Also, the high forces occurring during the reduction process increase the physical load on the surgeon preventing the reduction movements [9] and occasionally resulting in suboptimal fracture reduction [6].

Image guidance and planning, together with robotic assistance, can actually have a positive impact in overcoming the issues identified above, through enhanced 3D medical imaging and increased positioning accuracy.

**Fig. 1:**
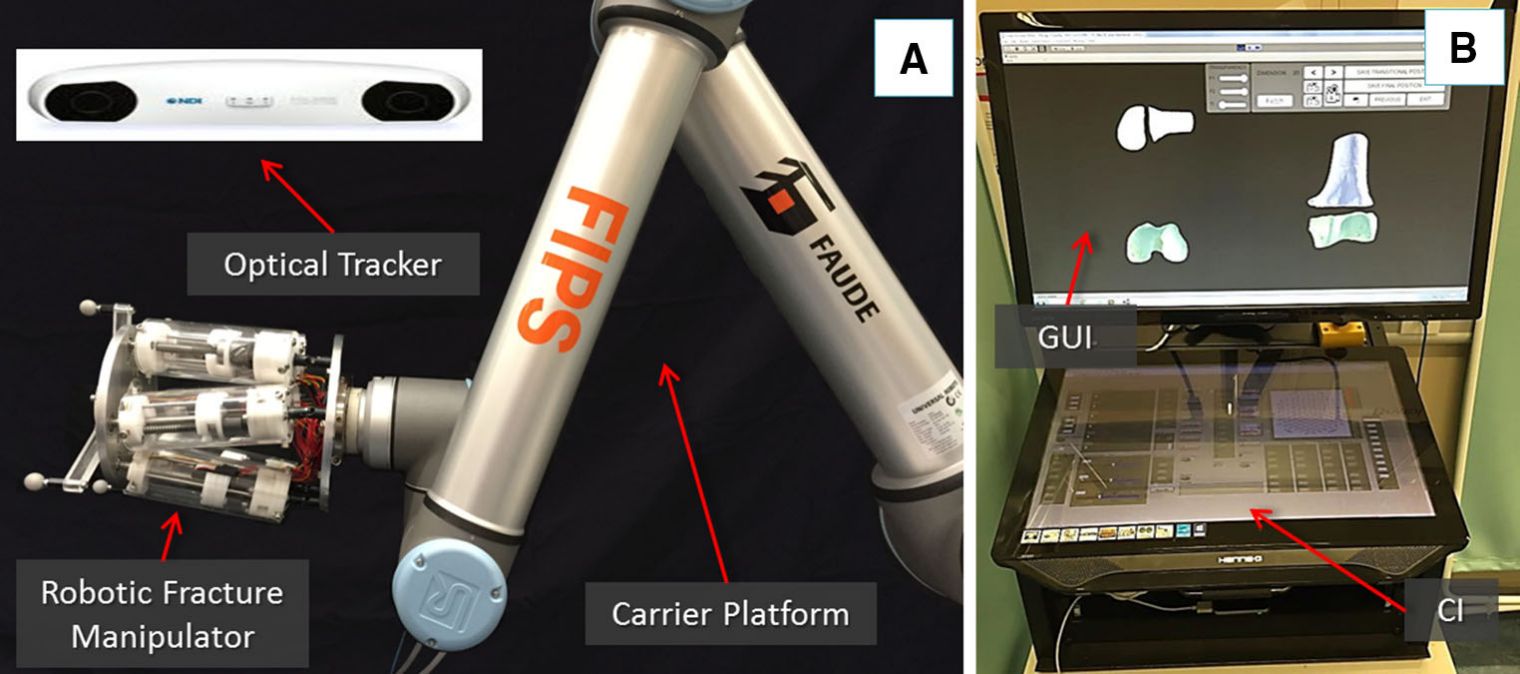
RAFS surgical system concept. The robotic fracture manipulator connected to the carrier platform, and the optical tracker (**a**); the system workstation running the GUI and the CI (**b**)

In this field, several studies have been carried out for long bone fracture reduction (specifically, femur shaft fractures) using 3D imaging. Joskowicz et al. [10] presented FRACAS, a computer-aided system that provides image guidance to the orthopaedic surgeon while reducing and fixing a long bone fracture. Fluoroscopic images are used to register pre-operative CT data to the intra-operative imaging. Warisawa et al. [11] developed a robotic system for femur fracture reduction, based on the orthopaedic traction table design (i.e. an operating table, which allows the application of a constant and adjustable pull [12]), using 3D CT image modelling for reduction path generation. Westphal et al. [13, 14] reported a robotic system for the reduction in femur shaft fractures based on a telemanipulated industrial serial robot. The surgeon controls the telemanipulated system from a console equipped with a joystick with force feedback to manipulate bone fragment attached to the robotic system based on 3D imaging data generated by intra-operative 3D fluoroscope. Tang et al. [15] and Graham et al. [16] utilized a parallel robot for the reduction in diaphyseal femur fractures based on 3D CT image reconstruction process for pre-operative planning. Buschbaum et al. [9] developed a system for computer-assisted repositioning of femoral fractures using 3D CT images. The system automatically generates the trajectories for reducing the fracture based on the computed surface curvature and fracture lines. In addition, a variety of computer-aided navigation systems using 2D fluoroscopic imaging were developed with the purpose of improving the reduction accuracy, such as [17–19]. However, all the described systems are restricted to long bone fractures, attempting to solve a different problem from intra-articular fractures that involve joints and typically require higher reduction accuracy [20]. Long bone fractures have smaller number of larger fragments that present a 2D problem for surgical reduction and are perceived easier to manage in the clinical setting using the current 2D imaging systems (fluoroscope). Intra-articular fractures are 3D fractures and are, therefore, more difficult to solve using 2D intra-operative images. Although some systems for fracture reduction based on 3D imaging are reported in the literature [9–11, 13–16, 21], their use has been limited to reduction in long bone fractures. To the best of our knowledge, no computer-assisted robotic system for intra-articular fracture reduction has been reported in the literature.

Robot-assisted fracture surgery (RAFS) is the focus of new research at Bristol Robotics Laboratory (BRL). Raabe et al. [22] developed the first robotic prototype for semi-automatic percutaneous reduction in intra-articular knee fractures using parallel robots for fragment manipulation. The key limitations of this system include the lack of closed-loop position control, no force feedback, limited operational workspace, the lack of intra-operative 3D imaging, and the need of intra-operative CT scan. This restricted the system’s reliability and usability in a real surgical environment. A second system prototype has been developed, introducing new robotic architecture and new control system strategy. The system is fully described in [23].

In this paper, we present a new navigation system that introduces pre-operative and intra-operative 3D guidance to reduce an intra-articular fracture using the robotic system developed at the BRL and described in [23]. This navigation system allows the surgeon to easily and precisely reduce the fracture by manipulating virtual models of the bone fragments generated by pre-operative CT data set. Orthopaedic manipulation pins are inserted into the bone fragments and tracked using a commercially available optical tracker (Polaris, NDI) through the attached optical tools (see Fig. 5). This allows the registration of the pre-operative data set with the patient in theatre, enabling a 3D intra-operative imaging and planning. The manipulation pins are connected to the robotic system [23], and the navigation system generates the motion commands to physically reduce the fracture based on the virtual reduction plan performed by the surgeon. This approach enables accurate intra-articular fracture reduction (robot-assisted) through small incisions (in a minimally invasive way), immediate evaluation of the reduction results (intra-operative 3D imaging), allowing, at the same time, an intra-operative modification of the pre-planned reduction strategy. The paper describes the new navigation system for robot-assisted intra-articular fracture surgery and evaluates its reduction accuracy through laboratory experiments on bone models.

**Table 1:** Fracture manipulation requirements [23]

Parameter	Value
Required translational accuracy	<1 mm
Required rotational accuracy	${<}5^{\circ }$
Translational and rotational workspace	2 mm–5 cm
	$5^{\circ }$–$180^{\circ }$
Forces/torques for manipulating fragments	$\sim$20 N
	$\sim$2 Nm

## Clinical requirements and surgical system configuration

### Clinical requirements

Clinical requirements were established through discussions with orthopaedic surgeons and analysis of various fracture cases [20], as described in  [23]. Distal femur fractures with fragment dislocations bigger than $5^{\circ }$ rotational and 1 mm translational displacements should be treated surgically. High-impact fractures can cause dislocations of more than 2 cm and $60^{\circ }$–$180^{\circ }$. During surgical reduction, the fracture fragments are typically approached through the anterior (front) of the limb $\pm 120^{\circ }$ from its vertical axis or from the lateral or medial side $\pm 60^{\circ }$ around the side axes of the limb. The required load capacity for the system has been defined by in vivo measured forces applied by surgeons during lower-limb surgical procedures. We instrumented a periosteal elevator and a traction table with two 6-DOF load cells, developed a dedicated data acquisition software, and analysed the force/torque data as reported in [12]. The procedures consisted of manipulating bone fragments using the instrumented device and collecting relative force/torque data. A summary of the clinical requirement is reported in Table 1.

### Surgical system configuration

The RAFS system used and improved upon in this research consists of the following components: a robotic fracture manipulator, a carrier platform, the system workstation, and the navigation system. The surgical system set-up is shown in Fig. 1n and its main subsystems are briefly described below. For an accurate description of the robotic system configuration (i.e. robot structure, workspace, kinematics, control strategy, and architecture), please refer to [23].

**Fig. 2:**
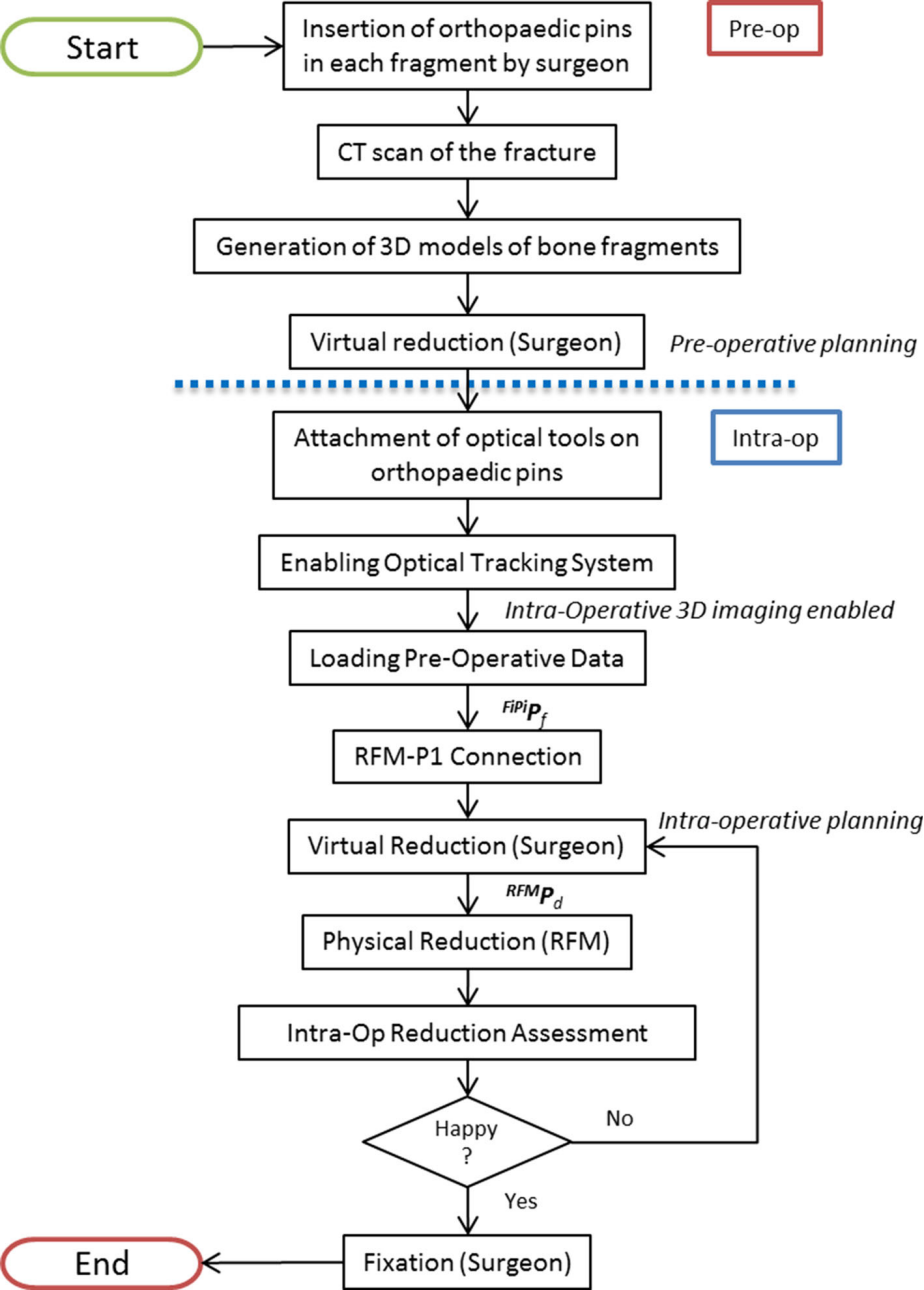
New clinical workflow for RAFS

*Robotic fracture manipulator* (*RFM*) This device (Fig. 1a), introduced in [24], is designed to be connected to the bone fragment through an orthopaedic pin for fragment manipulation. This component, based on parallel robot configuration with 6-DOF, has 6 motorized linear actuators fully computer-controlled and is able to realize accurate positioning within its workspace ($\pm \hbox {10.25 mm}$ along *x*, *y*, $\pm \hbox {15 mm}$ along z and rotational limits of $\pm 17^{\circ }$ around each axis). It provides a $0.03\pm \hbox {0.01 mm}$ translational accuracy and a $0.12^{\circ } \pm 0.01^{\circ }$ rotational accuracy [23]. The device mounts a 6-DOF force/torque load cell enabling a real-time force control. In order to fully cover the required operational workspace (Table 1; Fig. 2), the robotic manipulator is mounted on a carrier platform.

**Fig. 3:**
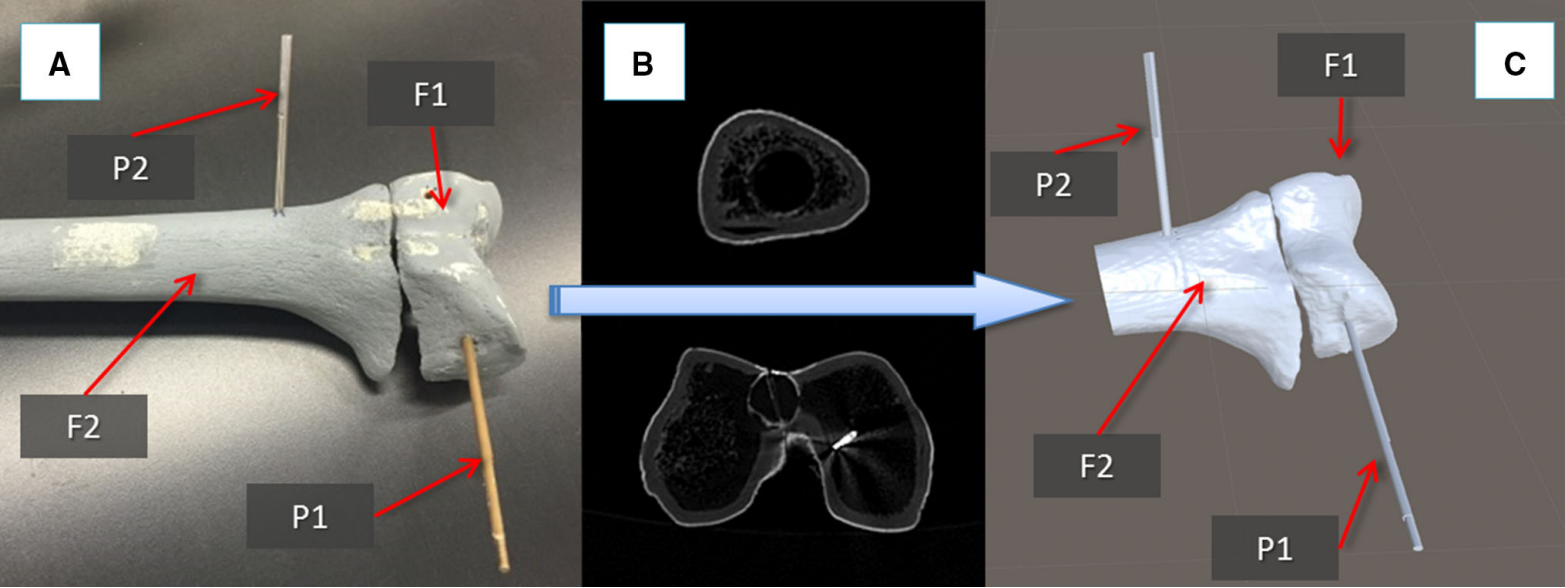
Fractured femur model with orthopaedic pins inserted (**a**), relative CT images (**b**), and 3D models (**c**)

*Carrier platform* (*CP*) This device (Fig. 1a) is used to position the RFM (which is connected as its end-effector) close to the orthopaedic pin. The CP provides an extended work space that can cover the required surgical workspace [23]. The RFM is then used to accurately manipulate the fragment to the desired, i.e. reduced, pose. The CP has 6-DOF, 3 translations and 3 rotations, covering the required operational workspace summarized above and described in [23].

*System workstation* It employs a host–target structure composed by a PC (host) and a real-time controller with FPGA (target), and a low-level motor controller. The host PC runs the graphical user interface (GUI) and the configuration interface (CI) (Fig. 1b). It creates the link between the surgical team and the robotic system. The GUI allows the surgeon to interact with the new navigation system, while the CI is used for system configuration and safety alarm messages. We adopted two separate screens: the GUI is displayed on a large 3D monitor dedicated to the surgeon, while the CI is displayed on a touch screen interface to allow a surgical assistant to change the settings configuration without requiring the surgeon’s intervention. The host PC communicates with the target controller via ethernet. The target controller (NI-compactRIO 9068, National Instruments) processes users’ commands and sends the motion commands to the low-level motor controller (EPOS 2 24/3, Maxon Motor) that executes the movement of the robotic system.

*Navigation system* This system, introduced in [25], consists of a reduction software, an optical tracking system, and a user controller. The reduction software receives pre-operative CT scan data of the fracture and generates the 3D models of the bone fragments. The GUI displays the 3D models and allows the surgeon to interact with them by using a controller for pre- and intra-operative planning of fracture reduction, i.e. virtual reduction. The optical tracking system (Polaris Spectra, NDI Inc.) provides a real-time (25 Hz) pose update of the optical tools (0.25 mm accuracy) connected to the bone fragments and the RFM. The optical tools have different and unique geometries to enable real-time tracking. The navigation system is described in the next section.

## Navigation system and system operation

This section describes the new navigation system for robot-assisted reduction in intra-articular fractures of the lower limb, along with a new clinical workflow (Fig. 2). This includes procedures for pre-operative virtual planning, intra-operative navigation, and physical reduction (using the robotic system) of the fracture. Complete two-part distal femur fractures (such as the one shown in Fig. 3) have been used for the development and the experimental validation of the proposed navigation system.

### Pre-operative planning

**Fig. 4:**
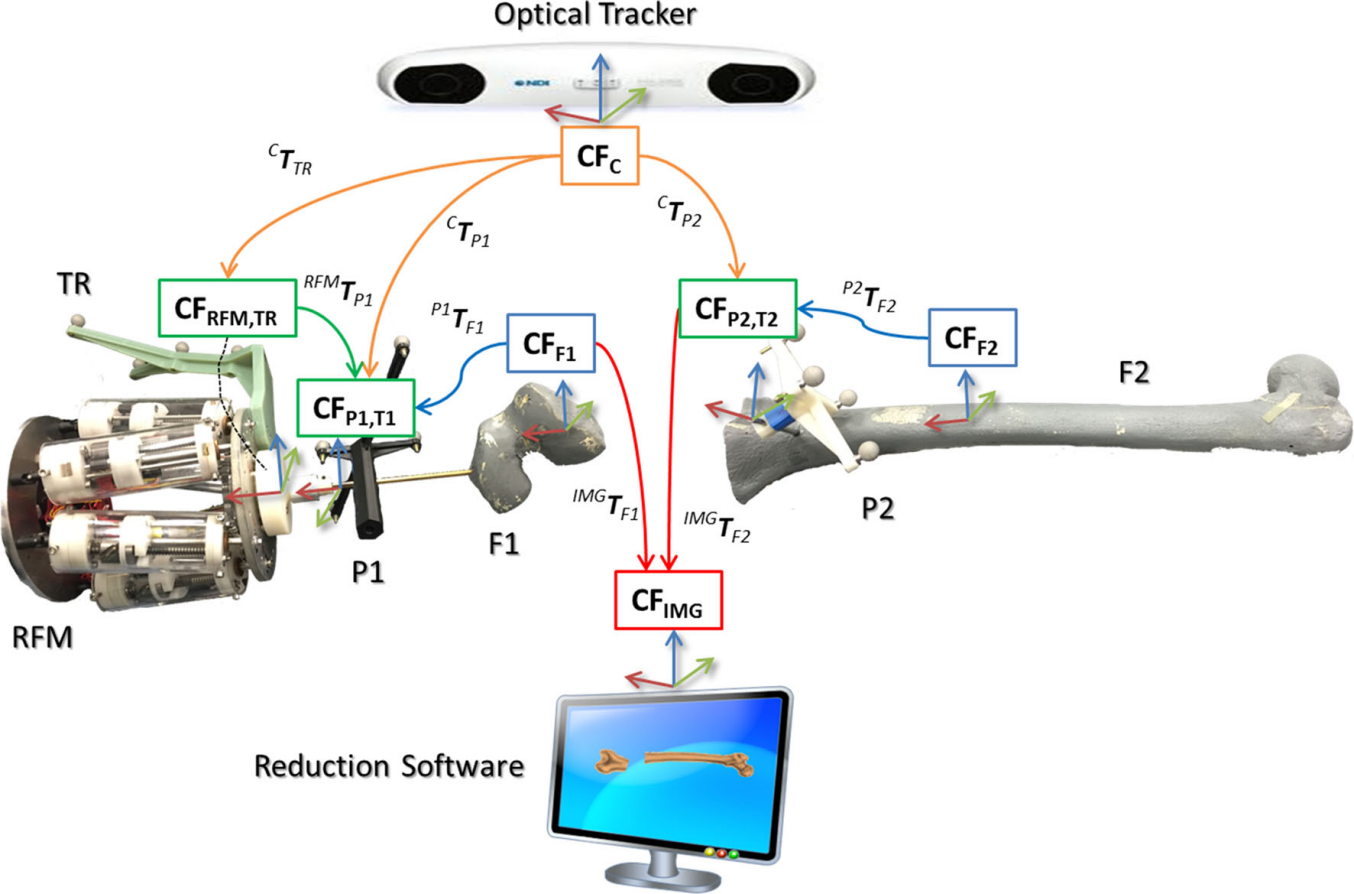
Components and transformations used in our navigation system

**Fig. 5:**
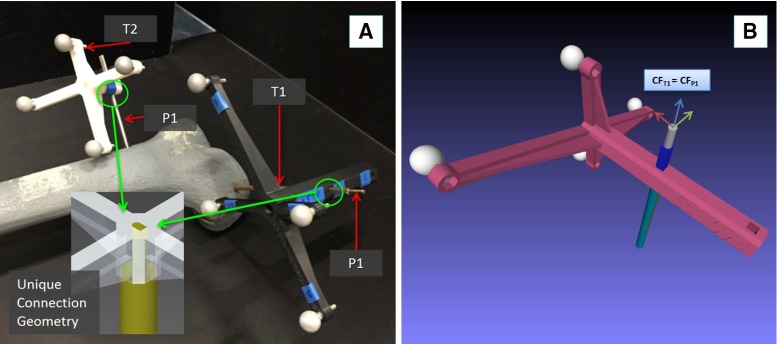
Optical tools T1 and T2 can be connected to their relative pins P1 and P2 in a unique way through a unique connection geometry (**a**); model of T1 inserted in P1: the coordinate frame of P1 $(\hbox {CF}_\mathrm{P1})$ is coincident with the coordinate frame of T1 $(\hbox {CF}_\mathrm{T1})$ (**b**). Similarly, $\hbox {CF}_\mathrm{P2}\equiv \hbox {CF}_\mathrm{T2}$

The procedure starts with the insertion of the orthopaedic pins into the bone fragments. Pin 1 (P1) is inserted in fragment 1 (F1), and pin 2 (P2) is inserted in fragment (F2), as shown in Fig. 3a. These pins will allow fragment manipulation through a small incision, i.e. minimizing the soft tissues damage. A pre-operative CT scan of the fracture and inserted pins is taken, and the resulting data set segmented to generate 3D models (STL format) of each bone fragment and the inserted pins using the ImageSim commercial software (Fig. 3b) [26]. These models are imported in the reduction software, and reference frames are defined as shown in Fig. 4: (1) The coordinate frame $\hbox {CF}_\mathrm{P1}$ is associated with P1, and the coordinate frame $\hbox {CF}_\mathrm{P2}$ is associated with P2. $\hbox {CF}_\mathrm{P1}$ and $\hbox {CF}_\mathrm{P2}$ are placed on the centre of the top end of the relative pin and oriented as shown in Fig. 5; (2) The coordinate frames $\hbox {CF}_\mathrm{F1}$ and $\hbox {CF}_\mathrm{F2}$ are associated with F1 and F2, respectively. $\hbox {CF}_\mathrm{F1}$, $\hbox {CF}_\mathrm{F2, }\hbox {CF}_\mathrm{P1}$, and $\hbox {CF}_\mathrm{P2}$ are measured in the CT image space and processed to get the homogeneous transformations $^{\mathrm{P1}}{{\varvec{T}}}_{\mathrm{F1}}$ and $^{\mathrm{P2}}{{\varvec{T}}}_{\mathrm{F2}}$ between P1–F1 and P2–F2, respectively [27]. $^{\mathrm{P1}}{{\varvec{T}}}_{\mathrm{F1}}$ and $^{\mathrm{P2}}{{\varvec{T}}}_{\mathrm{F2}}$ are considered to be constant during the operation. The surgeon virtually reduces the fracture using the reduction software GUI (described below) by manipulating F1 to match F2 (which is kept in a fixed pose) and generating the final poses for F1-P, i.e. $^{\mathrm{F1P1}}{\varvec{P}}_{f}$, in the reduced configuration. Results of the pre-operative procedure are stored in the system and used for intra-operative navigation, robot motion command calculation, and for the evaluation of the reduction results, as described in the next subsection.

### Intra-operative procedure

In the operating theatre, fragment F1 needs to be physically aligned to F2. This is accomplished using the robotic system described in the previous section. The robotic system is controlled by software according to the results of the pre- and intra-operative image analysis. The main components of the intra-operative procedure are the reduction software, the optical tracker, the robotic system, and the patient (i.e. the fracture). One optical tool (T1) is placed on the orthopaedic pin (P1) inserted in fragment 1 (F1), and a second optical tool (T2) is placed on the orthopaedic pin (P2) inserted in the reference bone (F2). A further optical tool (TR) is placed on the RFM (see Fig. 8a). The poses of the optical tools are measured in the optical tracking system ($\hbox {CF}_\mathrm{C})$, and the corresponding homogeneous transformations $^{\mathrm{C}}{{\varvec{T}}}_{\mathrm{TR}}$, $^{\mathrm{C}}{{\varvec{T}}}_{\mathrm{P1}}$, and $^{\mathrm{C}}{{\varvec{T}}}_{\mathrm{P2}}$ can be calculated. The orthopaedic pins P1 and P2 were designed to be connected in a unique way to the optical tools T1 and T2 (Fig. 5), having their coordinate frames coincident, i.e. $\hbox {CF}_\mathrm{P1 }\equiv \hbox {CF}_\mathrm{T1}$, and $\hbox {CF}_\mathrm{P2 }\equiv \hbox {CF}_\mathrm{P1}$. Therefore, assuming that $^{\mathrm{P1}}{{\varvec{T}}}_{\mathrm{F1}}$ and $^{\mathrm{P2}}{{\varvec{T}}}_{\mathrm{F2}}$ are constant during the operation, the optical tracker provides the actual poses of F1 (by tracking P1) and F2 (by tracking P2). This establishes a direct correspondence between the image space (reduction software, virtual models) and the task space (real fracture) by using the optical tracker, which enables the intra-operative imaging. This is described by the transformations $^{\mathrm{IMG}}{{\varvec{T}}}_{\mathrm{F1}}$ and $^{\mathrm{IMG}}{{\varvec{T}}}_{\mathrm{F2}}$.

**Fig. 6:**
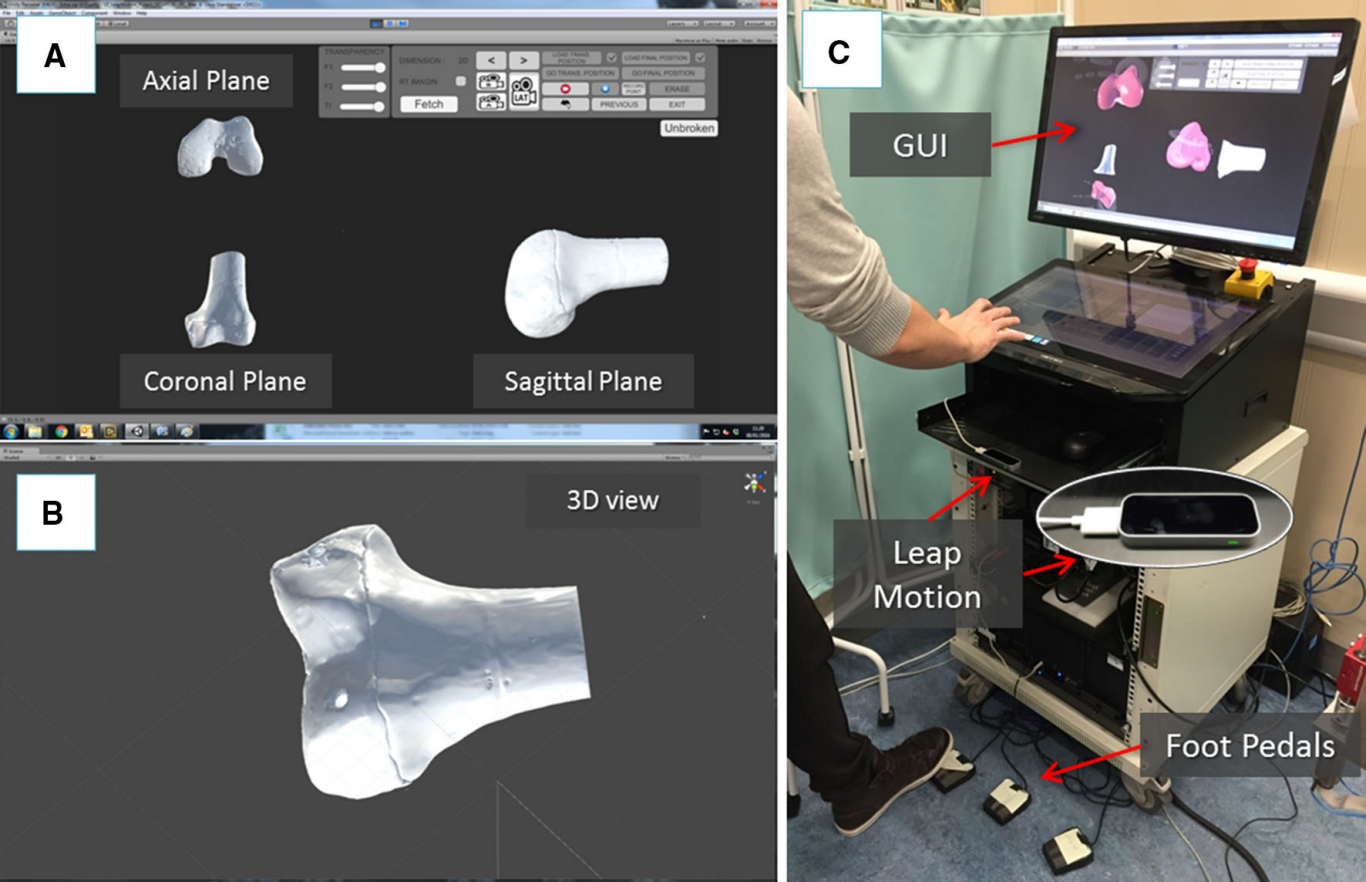
Reduction software GUI: 2D views according to the anatomical planes (**a**) and the 3D view (**b**) of the fracture; a user is virtually reducing the fracture interacting with the 3D models by using the leap motion and the foot pedals (**c**)

The next step consists in connecting the RFM to the fragment that has to be manipulated, i.e. F1 through P1. The system moves the CP in order to position the RFM close to the orthopaedic pin P1, whose pose in the physical space is provided by the optical tracker (through T1). An optical tracker TR is mounted on the RFM end- effector. The coordinate frame of TR is coincident with the coordinate frame of the robot end-effector, i.e. $\hbox {CF}_\mathrm{ROT} \equiv \hbox {CF}_\mathrm{EE}$. A surgeon’s assistant rigidly connects P1 to the RFM, and the reduction software—based on the relative position of P1 with respect to the RFM (by tracking TR)—calculates the transformation $^{\mathrm{RFM}}{{\varvec{T}}}_{\mathrm{P1}}$ between the robot and the orthopaedic pin P1.

Results of the pre-operative planning, i.e. the virtual reduction parameters, are uploaded into the intra-operative procedure, and the corresponding desired pose for the RFM to achieve the fracture reduction is computed as:

1 $$\begin{aligned} { }^{\mathrm{RFM}}P_d ={ }^{\mathrm{RFM}}T_{\mathrm{P1}} \times { }^{\mathrm{IMG}}T_{\mathrm{F1}}\times { }^{\mathrm{F1P1}}P_f \end{aligned}$$

Finally, the RFM executes the desired movement for F1 to achieve the physical reduction in the fracture, while reference bone F2 remains fixed. The real-time imaging updates the actual pose of the fragments in real time, and the surgeon can check intra-operatively the reduction in 3D without the use of any other intra-operative imaging device. If the reduction is acceptable, then the surgeon proceeds with the fixation of the fracture by using plate and screws or intramedullary nail, and the surgery ends.

**Fig. 7:**
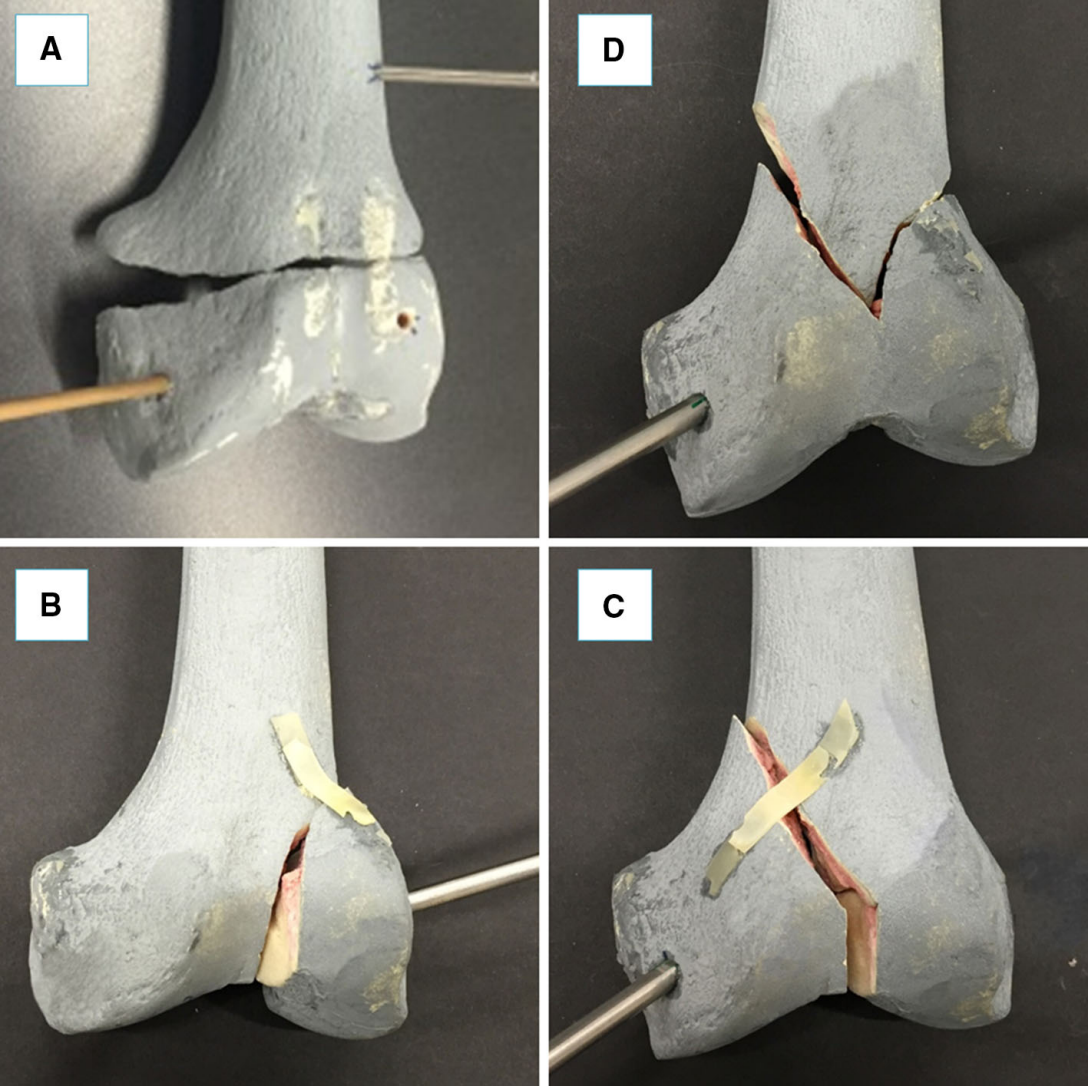
Distal femur fracture types used for the experimental evaluation of the system: simple fracture (**a**), lateral sagittal (**b**), medial sagittal (**c**), and articular Y-shape (**d**)

### Graphical user interface (GUI)

The reduction software runs a dedicated GUI (Fig. 6) developed using C# programming language on a Windows 7 PC, to allow the surgeon to interact with the navigation system. The GUI uses the freeware version of the Unity 5.1 engine [28] for the rendering, physics engine, and collision detection of the 3D models to simulate real-world condition in the virtual environment. A library for accessing the optical tracking system, robot, and controller was established. The GUI combines two separate modalities for pre-operative planning and intra-operative procedure. The pre-operative planning modality allows the surgeon to: (1) load and visualize the pre-generated models of the bones; (2) virtually interact with them; and (3) save the pre-operative planning results. The intra-operative procedure modality allows the surgeon to: (1) load and visualize the pre-generated models of the bones; (2) load the pre-operative planning results; (3) provide the actual position of the bones intra-operatively; (4) interact with the bones models, if still required; and (5) generate and send the motion command for the robotic system based on pre- and intra-operative imaging.

The GUI provides the surgeon with 2D views of each anatomical plane (i.e. sagittal, frontal, transverse [29]) and a 3D view of the fracture model (Fig. 6a). The 2D views (projections) of the fracture model allow the surgeon to perform a virtual reduction. The 3D view allows the surgeon to move the camera around the model in the virtual environment to assess the outcome of the reduction (Fig 6b).

The surgeon interacts with the 3D models through a contactless user controller to ensure the sterility of the whole procedure. The user controller chosen for this application is the Leap Motion [30], which is able to track and synthetize a 3D position and orientation of the hands in its workspace. Also, three foot pedals that provide on–off inputs to the system are included (1) to grab and release the fragment models, (2) to select a specific anatomical plane for interaction, and (3) to merge two fragments together that are further manipulated as one fragment (Fig. 6c).

## Experimental evaluation

**Fig. 8:**
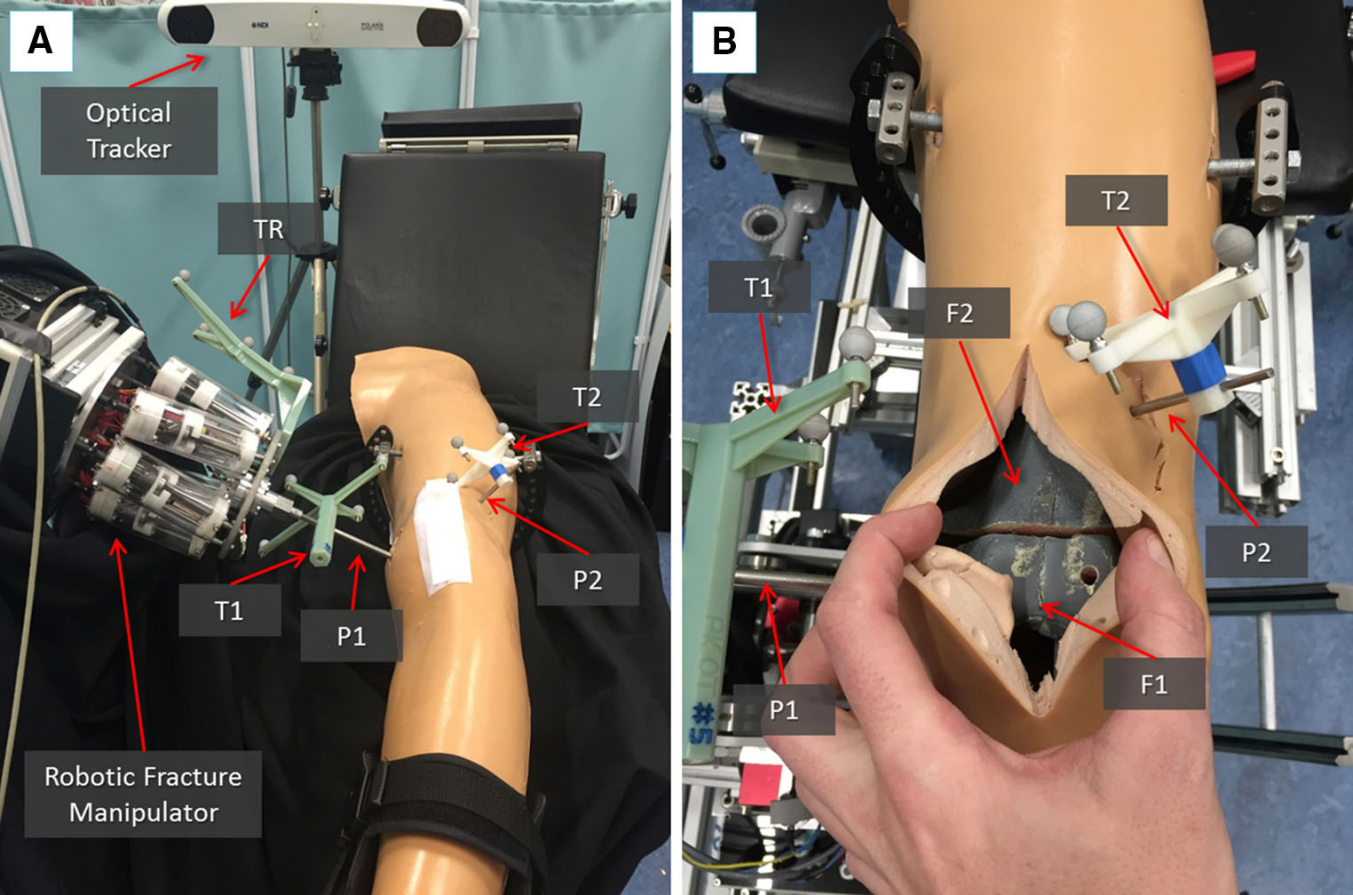
Experimental set-up: the robotic system is connected to the fracture fragment F1 through the orthopaedic pin P while P2 is inserted into the femur fragment F2 acting as a reference. The infrared camera tracks both the robot and the fragments through the optical tools TR, T1, and T2 (**a**); close-up of the fracture fragments and the inserted pins with optical tools (**b**)

The navigation system was tested performing 80 virtual reductions of 4 different 2-fragment distal femur fracture types (20 reductions for each fracture type), following the workflow described in the previous section. The distal femur fracture types chosen for the experimental evaluation (Fig. 7) were [5]: (1) simple fracture (33-A1), (2) lateral sagittal (33-B1), (3) medial sagittal (33-B2), and (4) articular Y-shape (33-C1). Also, 80 correspondent physical reductions were performed using the robotic system. A leg model has been manufactured ad hoc by Sawbones (Vashon Island, WA, USA). The leg includes solid-foam femur, patella, tibia, and fibula, encased in semi-flexible foam simulating the skin and the soft tissue surrounding the joint (i.e. muscles and flesh). Also, rubber bands were connected between the distal part of the femur and the proximal part of the tibia, in order to simulate knee ligaments (i.e. ACL, PCL, LCL, and MCL [31]). The experimental set-up is shown in Fig. 8.

### Pre-operative procedure

Two orthopaedic pins—P1 and P2—were inserted into the unbroken femur models: P1 in the distal part of the femur and P2 in the femur shaft (Fig. 5a). The relative pose of P1 with respect to P2 was obtained by temporarily placing two optical tools on the pins (T1 and T2 on P1 and P2, respectively) through the optical tracker. This relative pose, $^{\mathrm{F1P1}}{\varvec{P}}_{\mathrm{goal}}$, represents the ground truth for the reduction assessment, i.e. the target pose to reduce the fracture. The two optical tools T1 and T2 were removed from the pins.

The femurs were then fractured in two parts (see Figs. 3a, 8b), F1 and F2, maintaining the two orthopaedic pins inserted into their relative fragments and CT scanned. CT images were acquired pre-operatively with a SOMATOM Sensation 16 (Siemens Healthcare, Erlangen, Germany) CT scanner, with a voxel size of $\hbox {0.58 mm} \times \hbox {0.58 mm} \times \hbox {0.75 mm}$ and included the two fragments and the two inserted pins. 3D models of the fragments and the pins were generated using the ImageSim software and imported into the reduction software for the pre-operative surgical planning (see Figs. 3, 6). An orthopaedic surgeon was asked to virtually reduce each fracture 20 times, by manipulating F1 to match F2. Once the reduction is completed, the final (i.e. desired) pose $^{\mathrm{F1P1}}{\varvec{P}}_{f}$ of F1-P1 in the image space was stored.

### Intra-operative procedure

In the operating theatre, the fractured bone models with inserted pins were placed inside the leg model (Fig. 8). Optical tools F1 and F2 were placed again on P1 and P2, respectively, and the optical tracker turned-on, enabling the intra-operative imaging and showing the actual pose of the two bone fragments in the GUI. The CP positioned the RFM close to P1, which was then connected to the RFM, as described in the previous section. Results from the pre-operative planning ($^{\mathrm{F1P1}}{\varvec{P}}_{f})$ were imported into the intra-operative procedure, and the reduction software calculated the desired pose $^{\mathrm{RFM}}{\varvec{P}}_{d}$ for the RFM in the task space using equation (1). Finally, the robot executed the physical reduction, and the actual pose of F1-P1 ($^{\mathrm{F1P1}}{\varvec{P}}_{a}$) after the reduction was measured by the optical tracker.

### Evaluation metrics and results

During the experiments described above, the final poses of F1-P1 after the pre-operative virtual reduction ($^{\mathrm{F1P1}}{\varvec{P}}_{f})$ and after the intra-operative physical reduction using the robot ($^{\mathrm{F1P1}}{\varvec{P}}_{a})$ of each fracture were saved for subsequent comparison with the desired pose for F1-P1 in its unbroken configuration ($^{\mathrm{F1P1}}{\varvec{P}}_{\mathrm{goal}}$). These comparisons allowed the objective evaluation of the surgical system accuracy, measured as: (1) virtual reduction accuracy, and (2) physical reduction accuracy. The metrics chosen for the system accuracy evaluation were the root-mean-squared error (RMSE), and the maximum absolute error (MAE) measured during both virtual and physical reductions. Also, the time employed to complete each reduction (both virtual and physical) was recorded as a system performance evaluation metric. Finally, the average load applied during the physical reduction was calculated to analyse the contact forces and torques between the RFM and the leg (i.e. bones and soft tissues).

**Fig. 9:**
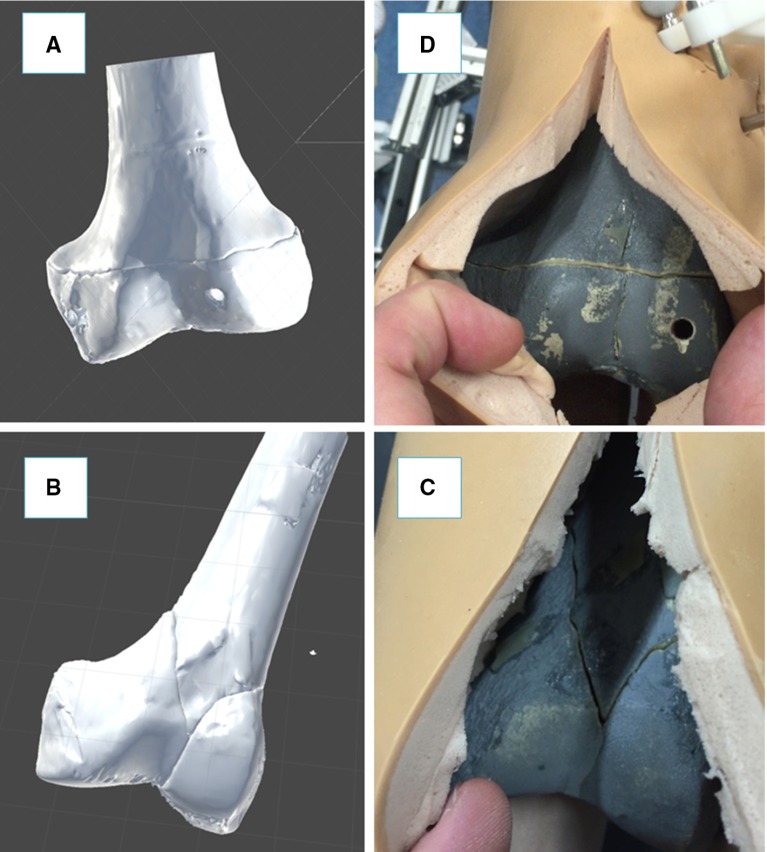
Experimental validation results examples: virtual reduction in the fracture performed by the surgeon on a simple fracture (**a**) and on an articular Y-shape fracture (**b**); correspondent physical reduction achieved by the robotic system, (**c**, **d**) respectively

Results from evaluation experiments are reported in Table 2 (virtual reduction) and Table 3 (physical reduction), while visual reduction examples are shown in Fig. 9.

**Table 2:** Results—virtual reduction

Fracture type	Number of reductions	RMSE	MAE	Reduction time (s)
Metaphyseal fracture	20	$0.95 \pm \hbox {0.3 mm}$	1.03 mm	$73.4 \pm 12.7$
(33-A1)		$1.02^{\circ }\pm 0.1^{\circ }$	$1.15^{\circ }$	
Lateral sagittal	20	$0.83 \pm \hbox {0.13 mm}$	0.96 mm	$79.8 \pm 22.8$
(33-B1)		$0.89^{\circ }\pm 0.3^{\circ }$	$1.38^{\circ }$	
Medial sagittal	20	$0.86 \pm \hbox {0.25 mm}$	1.3 mm	$93.9 \pm 51.3$
(33-B2)		$1.02^{\circ }\pm 0.33 ^{\circ }$	$1.5^{\circ }$	
Complete articular	20	$0.94 \pm \hbox {0.1 mm}$	1.5 mm	$134.2 \pm 55.9$
(33-C1)		$1.4^{\circ }\pm 0.5^{\circ }$	$3.15^{\circ }$

**Table 3:** Results—physical reduction

Fracture type	Number of reductions	RMSE	MAE	Reduction time (s)	Applied load
Metaphyseal fracture	20	$1.03 \pm \hbox {0.2 mm}$	1.04 mm	$74.8 \pm 2.5$	16.2 ± 1.7 N
(33-A1)		$1.19^{\circ }\pm 0.1^{\circ }$	$1.2^{\circ }$		1.3 ± 0.3 Nm
Lateral sagittal	20	$0.91 \pm \hbox {0.9 mm}$	1.0 mm	$75.3 \pm 2.1$	16.5 ± 1.9 N
(33-B1)		$1.03^{\circ }\pm 0.3 ^{\circ }$	$1.4^{\circ }$		1.5 ± 0.5 Nm
Medial sagittal	20	$0.96 \pm \hbox {0.3 mm}$	1.35 mm	$76.1 \pm 2.4$	16.1 ± 1.5 N
(33-B2)		$1.19^{\circ }\pm 0.3^{\circ }$	$1.55^{\circ }$		1.4 ± 0.4 Nm
Complete articular	20	$1.04 \pm \hbox {0.2 mm}$	1.53 mm	$75.9 \pm 2.3$	16.7± 1.6 N
(33-C1)		$1.56^{\circ }\pm 0.6 ^{\circ }$	$3.2^{\circ }$		1.58 ± 0.7 Nm

## Discussion

This study introduced a new navigation system for pre-operative reduction planning and intra-operative 3D guidance of intra-articular fractures using the robotic system developed at the Bristol Robotics Laboratory [23]. The results from the experimental validation trials demonstrated that the proposed navigation system created for the RAFS system is able to meet the reduction accuracy requirements for joint fracture surgeries, i.e. 1 mm and $5^{\circ }$ (Table 1). The metrics chosen for the system accuracy evaluation, i.e. RMSE and MAE, are strictly related to the operational safety and efficiency of the surgical system. In general, high values of RMSE and MAE give an account of how far the manipulated fragment is from the desired, i.e. reduced, position, and physical reduction procedures.

The navigation system allowed the surgeon to virtually reduce all the fractures with a maximum residual positioning error (RMSE) lower than 1 mm, $5^{\circ }$ (clinical requirements, Table 1). The best result was obtained reducing lateral sagittal fractures (33-B1) with a residual positioning error (RMSE) of $0.83 \pm 0.13\,\hbox {mm}$ and $0.89^{\circ }\pm 0.3^{\circ }$. A similar result was obtained reducing medial sagittal fractures (33-B2), as shown in Table 2. Metaphyseal fractures (33-A1) and complete articular fractures (33-C1) resulted more challenging with a residual positioning error (RMSE) of $0.95\pm \hbox {0.3 mm}$ and $1.02^{\circ }\pm 0.1^{\circ }$, and $0.94 \pm \hbox {0.1 mm}$ and $1.4^{\circ }\pm 0.5^{\circ }$, respectively. The correspondent physical reduction accuracies are reported in Table 3. This data demonstrate that the RAFS system is able to meet the clinical requirements of 1 mm, $5^{\circ }$ presenting a maximum residual positioning error (RMSE) of $1.04 \pm \hbox {0.2 mm}$ and $1.56^{\circ }\pm 0.6^{\circ }$ (complete articular fractures), when the robot reduced the fractures. This result is achieved thanks to the sub-millimetre positioning accuracy of the robotic system, which is 0.09 mm and $0.15^{\circ }$ as demonstrated in [23]. Moreover, the measured MAEs further demonstrated that the system permits excellent reduction accuracies (both virtual and physical), helping the surgeon to avoid large deviations from the desired reduction. Results demonstrated that the residual inaccuracies are mainly due to the virtual reduction procedure rather than the physical one. This can be further improved by creating 3D virtual models of the bones from CT data with a better resolution, i.e. using a high-resolution CT scanner. However, the experiments also demonstrated that the proposed system has a higher reduction accuracy when compared with other systems based on 3D imaging reported in the literature such as [9–11, 13–15, 21]. The automated reduction system for femur fractures proposed by Buschbaum et al. [9] resulted in a residual reduction error of $1.2 \pm \hbox {0.9 mm}$ and $2.6^{\circ }\pm 2.8^{\circ }$. This level of accuracy could be sufficient for femur shaft reduction applications, but it may not be sufficient for fractures that involve joints. Even though an automatic reduction could be more efficient, we believe that the surgeon should be in full control of the system during the surgery. The FRACAS system proposed by Joskowicz et al. [10] for long bone fracture reduction and fixation uses 3D models generated by pre-operative CT data and one intra-operative 2D fluoroscopic image to guide the surgeon in reducing and fixing a fracture. The system potentially decreases the level of radiation exposure to the surgeons (only one intra-operative fluoroscopic image is required) and results in a sub-millimetre registration accuracy between 2D and 3D images. However, only the accuracy of image registration and calibration has been assessed, while the physical fracture reduction accuracy evaluation is not shown. The automated traction table proposed by Warisawa et al. [11] presented an average positioning error of only 0.57 mm and $0.12^{\circ }$, but it seems applicable only to shaft fractures given its non-invasive attachment to the patient’s foot. The system proposed by Westphal et al. [13, 14] presented a reduction displacement of about 2 mm and $2.9^{\circ }$ on femur shaft reductions, which is not sufficient for intra-articular fractures. Wang et al. [21] designed a parallel robot mechanism to reduce femur shaft fractures with an accuracy of 2.43 ± 0.49 mm (lateral translation) and $2.26^{\circ }\pm 0.23^{\circ }$ (angulation), which is, again, not acceptable for joint fractures. A similar system from Tang et al. [15] resulted in a residual deviation of 1.24 ± 0.65 mm for the axial deflection, $1.19 \pm 0.37\,\hbox {mm}$ for the translation, $2.34^{\circ } \pm 1.79^{\circ }$ for the angulation, and $2.83^{\circ } \pm 0.9^{\circ }$ for the rotation (on bovine femur shaft fractures). However, this system requires a CT scan of both limbs (both injured and healthy side) and the connected robot, and lacks intra-operative real-time image guidance.

The average time the surgeon took to virtually reduce 80 fractures using the navigation system is about 95 seconds. Similarly, the robot employed on average about 75 seconds to physically reduce the fracture based on the virtual reduction. Therefore, the entire reduction procedure can be accomplished in about 3 minutes, arguably speeding up the entire fracture surgery.

The accuracy of the navigation system—and in particular the virtual reduction procedure—can only be affected by the accuracy of the segmentation of the CT data set and not by the actual specimen being scanned (e.g. human bone vs. Sawbones) [9]. However, human bones are surrounded by soft tissue which generates forces and torques on the robotic manipulator during the physical reduction. Therefore, the evaluation experiment has been conducted on an artificial phantom simulating the bones and the soft tissue. The loads measured during the physical reductions resulted in average force of about 16.3 N and average torque of 1.4 Nm (Table 3). The average loads measured during the trials are comparable (slightly lower, due to the absence of real soft tissues in our model) than the loads measured during experiments conducted on ex vivo animal specimen [23], and during real fracture surgeries [12, 32]. The load measured during the reduction in different fracture types is roughly the same, which shows that it does not depend on the shape of the fracture but on the contact between the manipulation pins and the leg model. This is also an indicator of correct reduction trajectories for different fracture types, i.e. the manipulated fragments smoothly reach the desired positions.

## Conclusion

In this paper, we presented a navigation system which allows the surgeon to virtually reduce bone fractures, i.e. distal femur fractures. The motion commands generated by the navigation system are sent to our robotic system which physically reduces the fracture.

The bone fragments are segmented from a pre-operative CT scan, and 3D virtual models are generated. Fragments are visualized on the screen and can be virtually manipulated through the dedicated contactless GUI. The fracture is reduced by moving the fragments to the desired target position, thereby completing the pre-operative planning procedure. During the surgery, the actual position of the bone fragments is provided by an optical tracker enabling real-time 3D imaging. The surgeon monitors the reduction process and can correct and modify the virtual reduction intra-operatively if required. The motion commands for the robot connected to the bone fragment are generated, and the fracture physically reduced based on the surgeon’s virtual reduction using the navigation system.

Experimental outcome demonstrates the accuracy and effectiveness of the proposed navigation system, presenting a fracture reduction accuracy of about 1 mm and $1.5^{\circ }$—when used in conjunction with our robotic system—meeting the clinical requirements for distal femur fracture reduction procedures.

In summary, the major advantages of the proposed system are as follows: (1) enhanced 3D visualization required to better understand the three-dimensional fracture configuration; (2) the reduction strategy can be accurately pre-planned by the surgeon; (3) immediate evaluation of the reduction results through the real-time 3D guidance; and (4) accurate and safe robotic assistance for the physical reduction in the fracture with minimized soft tissue damage (minimally invasive approach) for a better clinical outcome. The actual hardware configuration allows the physical reduction in only one fragment at the time. In the next step of development, a second robot (CP + RFM) will be included in the system to allow simultaneous manipulation of two fragments. This will allow treatment for other types of distal femur fractures (e.g. multi-fragmented), but also fractures of other joints, e.g. pelvis, ankle, neck of femur, and upper-limb joints.

Further studies are planned in the optimization of the navigation system through the implementation and evaluation of different user controllers which can potentially further improve the virtual reduction accuracy of the system. Usability study with experienced surgeons is also planned to evaluate the performance of the navigation system, gathering not only objective measurements from the surgeons’ performance using the system but also their subjective perception of it. Moreover, cadaveric trials will be shortly conducted.

## Electronic supplementary material

Below is the link to the electronic supplementary material.

Supplementary material 1 (avi 2184 KB)

Supplementary material 2 (avi 5870 KB)

Supplementary material 3 (avi 2500 KB)
